# Whole-Genome Resequencing Identifies Candidate Genes for Tail Fat Deposition in Sheep

**DOI:** 10.3390/ani15203046

**Published:** 2025-10-20

**Authors:** Xiaowen Zhang, Yufei Li, Yongqing Zhao, Penghui Guo, Yong Cai, Hongwei Xu, Xin Cao, Qiongyi Li, Xiaoxia Ma, Derong Zhang, Jialin Bai

**Affiliations:** 1 Biomedical Research Center, Northwest Minzu University, Lanzhou 730030, China; 1073324010071@st.gsau.edu.cn (X.Z.); maxiaoxia956@163.com (X.M.); 2 College of Veterinary Medicine, Gansu Agricultural University, Lanzhou 730070, China; 3 College of Life Science and Engineering, Northwest Minzu University, Lanzhou 730030, China; zyqbd@163.com (Y.Z.); skyguoph@xbmu.edu.cn (P.G.); xuhongwei@xbmu.edu.cn (H.X.); caoxin-juliet@163.com (X.C.); lqy@xbmu.edu.cn (Q.L.); 4 Gansu Food Inspection Institute, Lanzhou 730030, China; lyf4718@163.com; 5 Experiment Teaching Department, Northwest Minzu University, Lanzhou 730030, China; caiyong1979@163.com

**Keywords:** sheep-tailed, whole genome resequencing, selection signal, lipid metabolism

## Abstract

**Simple Summary:**

It is evident that caudal fat deposition demonstrates considerable interbreed variation. The Lanzhou fat-tailed sheep, a Chinese indigenous long-fat-tailed breed, has experienced a precipitous decline in population due to urbanization and market-driven preference for lean meat. In contrast, the Hu sheep exhibits a short-fat-tailed phenotype. Even though a considerable number of studies have investigated selection signatures associated with ovine tail morphology, genomic research on long-fat-tailed breeds remains limited. In order to address this gap, whole-genome resequencing was employed in combination with F_ST_, XP-EHH, and XP-CLR analyses. This was undertaken in order to identify selection signatures that differentiate long- and short-fat-tailed breeds. The present study has revealed *DAB1* and *GPC5* to be shared candidate genes, thus demonstrating their synergistic involvement in lipid metabolism regulation across both breeds.

**Abstract:**

Excessive adipose tissue accumulation in sheep disrupts insulin signaling, inducing insulin resistance, and alters energy partitioning mechanisms. These changes adversely affect both ovine health and production efficiency. This study employed whole-genome resequencing to conduct selection signal analysis in long-fat-tailed (Lanzhou fat-tailed sheep) and short-fat-tailed (Hu sheep) breeds, investigating the genetic basis underlying divergent lipid metabolism-related traits between these distinct tail phenotypes. Fifteen healthy adult individuals, each from long-fat-tailed (Lanzhou Large-tailed sheep) and short-fat-tailed (Hu sheep) breeds, underwent whole-genome resequencing. Whole-genome resequencing analyses via F_ST_, XP-CLR, and XP-EHH identified 75 significantly selected regions (*p* < 0.01), revealing eight key candidate genes (*DAB1*, *DPP10*, *EPHA6*, *GPC5*, *KLF12*, *PAK7*, *PTPN3*, *TENM3*). Subsequent functional enrichment analysis demonstrated significant enrichment of *DAB1* and *GPC5* in lipid metabolic processes (GO:0006629). Employing whole-genome resequencing-based selection signal analysis in long-fat-tailed (Lanzhou Large-tailed sheep) and short-fat-tailed (Hu sheep) breeds, this study identified two key lipid metabolism-associated genes (*DAB1* and *GPC5*). These findings provide critical insights for conserving genetic resources and informing molecular breeding strategies targeting divergent tail phenotypes.

## 1. Introduction

In China, indigenous sheep breeds are classified into five categories based on tail morphology: short thin-tailed, long thin-tailed, short fat-tailed, long fat-tailed, and fat-rumped varieties [1,2,3]. Thin-tailed sheep represent the ancestral type, with fat-tailed breeds emerging later through natural and artificial selection [4,5,6]. Fat tails serve as energy reservoirs during periods of nutritional stress, particularly in arid or cold climates [1,5,6]. Historically, tail fat provided a critical food source, though modern dietary trends have reduced its desirability [7]. Additionally, excessive fat storage is now seen as metabolically costly in commercial sheep farming, reducing feed efficiency and potentially interfering with reproduction due to physical impediments during mating [8].

In indigenous Ethiopian sheep, there is a positive correlation between tail width and girth, and fat accumulation, indicating that breeds with longer tails and shorter tails tend to exhibit higher fat deposition levels [9,10,11]. Scholars have made significant advancements in multi-omics technology concerning sheep tail fat deposition. Genomic studies have identified crucial genes and quantitative trait loci (QTLs) linked to this process. Specifically, *PDGF-D* polymorphisms influence tail fat development by modulating transcription factor binding [1], while *SREBF1*/*SREBF2* play a dominant role in lipid synthesis [12]. Additionally, *IL-6* stimulates lipolysis via the TNF/MAPK signaling pathway [13], and SNPs of *TRAPPC9* and *BAIAP2* genes are associated with lipid storage efficiency [14,15]. Recent multi-omics studies have identified key genes and regulatory pathways (e.g., PPAR, AMPK/HIF-1) influencing tail fat deposition, including both protein-coding and non-coding RNAs [3,5,7,13,15,16,17].

The Lanzhou fat-tailed sheep (LLS), indigenous to Lanzhou City and adjacent loess plateau gully regions in Gansu Province, represents a distinctive Chinese long-fat-tailed breed. Its caudal adipose tissue constitutes 15–20% of body mass, serving as a critical energy reservoir [16,17]. The decline of the population size of LLS may be related to the decline of genetic diversity. It is therefore evident that genomic research provides a scientific basis for the protection of LLS, and that this in turn helps to formulate targeted conservation measures. Hu sheep (HS), an indigenous Chinese breed primarily distributed in the Taihu Lake basin of the Yangtze River Delta, is classified as a short-fat-tailed sheep variety [18]. Contemporary Chinese sheep production systems increasingly employ HS as the dam line in strategic crossbreeding with exotic meat breeds. As the preferred genotype for intensive commercial hybridization systems, HS contributes significantly to genetic improvement and productivity in China’s meat sheep sector.

Whole-genome resequencing (WGR) is a molecular biology technique considered efficient and accurate [19,20]. Research shows that systematically applied F_ST_, XP-EHH, and XP-CLR analyses, thereby revealing genetic variations and selection signals associated with wool traits in long-fat-tailed sheep. The findings of the present study provide a valuable framework for the genetic improvement of ovine species [21]. Research shows that investigated the population genetic structure and selection signatures in long-fat-tailed sheep using F_ST_ and XP-CLR analyses. Integration of XP-EHH was utilized to assess genetic diversity in genomic regions, elucidating molecular mechanisms underlying adaptation to extreme environments in this breed [22]. Divergent selection between coarse-wool and fine-wool sheep breeds was assessed via XP-EHH, and targeted genes that significantly influence wool quality were identified, notably *PRX* (peroxidase) and *TCF3* (hair follicle stem cell regulator) [23]. While genomic selection analyses have been widely applied in cattle and wool sheep, their use in understanding tail fat regulation in sheep remains limited. To address this, the study performed whole-genome resequencing on LLS and HS. Using F_ST_, XP-EHH, and XP-CLR analyses, we identified genomic regions under selection in long- versus short-fat-tailed breeds. This approach sheds light on the gene regulatory mechanisms underlying fat deposition in fat-tailed sheep, providing a theoretical foundation and technical support for conserving genetic resources, improving breeds, and advancing molecular breeding.

## 2. Materials and Methods

### 2.1. Sample Collection

This study selected 15 healthy adult LLS, from the Taiji Town, Sanmatai Breeding Farm, Yongjing County, Gansu Province, China) and 15 HS (Hunan sheep, from the Purebred Hunan Sheep Breeding Farm, Minqin County, Gansu Province, China) sheep (Table 1). Blood was collected from the jugular vein of each sheep (2 mL), added to EDTA and stored at −20 °C for genomic DNA extraction. All animal experiments were approved by the Ethical Committee of Experimental Animal Center of Biomedical Research Center of Northwest Minzu University in compliance with the National Guidelines for Experimental Animal Welfare (Approval No. xbmu—sm—2025100, 11 August 2025).

### 2.2. DNA Extraction and Sequencing Library Construction

Total DNA was extracted from blood using a genomic DNA extraction kit (NMG0161, Naming Magnetism, Wuhan, China). The purity and concentration of the DNA were assessed with a Qubit 3.0 fluorometer (Invitrogen, Carlsbad, CA, USA). The integrity of the genomic DNA was evaluated through agarose gel electrophoresis. Sequencing was conducted by Wuhan Aiki Baike Biotechnology Co., Ltd. (Wuhan, China), utilizing the Illumina HiSeq 2500 platform (Illumina, San Diego, CA, USA) with a PE125 sequencing strategy at a depth of 10x. The raw sequencing reads were stored in FASTQ file format for subsequent analysis.

### 2.3. Quality Control and Comparison

The filtering steps were as follows: (1) reads containing junctions were removed, retaining only the remaining reads; (2) reads with an N proportion exceeding 10% were eliminated; (3) low-quality reads were discarded, defined as those where the number of bases with a quality value of Q ≤ 20 constituted more than 50% of the entire read. High-quality sequencing data were aligned to the sheep reference genome using the MEN algorithm of BWA (version 0.7.15), with the alignment parameter set to -k 32 -M. The alignment results in SAM format were converted to BAM format using SAMtools (Version: 1.3.1). Coverage was subsequently calculated using Bedtools (version 2.25.0) after marking duplicate reads with Picard (version 2.18.7) (http://sourceforge.net/projects/picard/ (accessed on 1 July 2025)). Variants were functionally annotated using ANNOVAR software 86 (version release of 16 April 2018). To enhance the accuracy of data analysis, raw SNPs were filtered, and the high-quality SNP markers that passed the filtering criteria were utilized for selection signal analysis. The filtering criteria included the removal of marker loci with a deletion rate greater than 20% and a minimum allele frequency (MAF) of no less than 5%.

### 2.4. Selection Signal Analysis

Selection signal analysis was conducted using three methods: F_ST_, XP-CLR, and XP-EHH. Differences in allele frequencies between populations were calculated using the F_ST_ method, which identified regions subjected to selection among populations. Selection signals within populations were detected using the XP-CLR and XP-EHH methods to screen for gene regions under selection. XP-CLR is a linkage disequilibrium-based method for detecting selection signals that identifies genomic regions under selection by comparing differences in allele frequencies and linkage disequilibrium patterns across different populations. XP-EHH, on the other hand, is an extended haplotype-based method for detecting selection signals, which identifies gene regions under positive selection by analyzing the distribution of haplotypes within a population.

### 2.5. Detection and Annotation of Candidate Genes

The F_ST_ values calculated were statistically analyzed and Manhattan distribution plots were drawn. The top 1% SNPs were selected as selected loci. The selected loci were annotated using NCBI (National Center for Biotechnology Information) database and CSIRO database Ovis aries 3.1 genome database. The core SNP of the selection signal generation region was taken as the center, and the upstream and downstream were extended by 50 kb as the selection region, and the genes falling in this selection region were defined as the “candidate genes” of the selection signal. Analyzing the basic information of candidate genes, including gene location, encoded protein, involved biological process and molecular function, lays a foundation for further study of gene function and mechanism.

### 2.6. Candidate Gene Enrichment Analysis

Using DAVID v6.8 (Database for Annotation, Visualisation and Integrated Discovery https://davidbioinformatics.nih.gov/home.jsp (accessed on 17 October 2025) database, the candidate genes were subjected to GO (Gene-Ontology) functional enrichment analysis. GO (Gene Ontology) function enrichment analysis and KEGG (Kyoto Encyclopedia of Genes and Genomes) pathway analysis were performed on the candidate genes using DAVID v6.8 (Database for Annotation, Visualisation and Integrated Discovery) database. The GO functional enrichment analysis mainly includes three levels: molecular function, biological process and cellular component.

## 3. Results

### 3.1. Overview of Sequencing Data

In this study, the DNA samples of 15 LLS and 15 HS were sequenced and quality controlled. First, low-quality paired ends were removed, resulting in high-quality paired end reads from 30 samples with a mass of 97.85% for Q20 and 93.26% for Q30 bases (Table S1). A total of 414,823,855 SNPs were identified from the genome of 30 samples with an average sequencing depth of 10× for further analysis. After DNA sample sequencing and quality control in the early stage, 3294097496 and 4175193938 clean reads were obtained in the resequencing results of 15 LLS and 15 HS, respectively, for later mutation detection. After a series of alignment, mutation detection and quality control, 1,043,070 SNPs were identified on 26 autosomes +1 sex chromosome for further genomic genetic analysis.

### 3.2. Population Genetic Analysis

**Figure 1:**
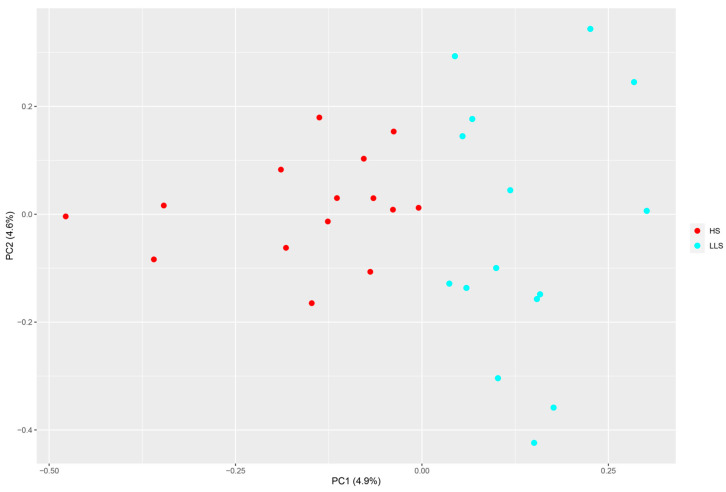
Analysis of the genetic structure of the population: principal component analysis (PCA) (red color represents a Hu sheep population, and blue color represents a Lanzhou fat-tailed sheep population).

We conducted population genetic analysis of two groups (HS and LLS) using high-quality SNP data to understand the genetic relationships and differences between these groups. The PCA (principal component analysis) plot (Figure 1) shows that PC1 and PC2 explain 4.9% and 4.6% of the genetic variation, respectively. The plot illustrates the clustering of the two groups, with their distribution in genetic space being relatively close; among them, the HS and LLS groups form distinct clusters.

### 3.3. Identification of Signatures of Selection Between Long-Fat-Tailed and Short-Fat-Tailed Sheep Breeds Using *F_ST_* Analysis

**Figure 2:**
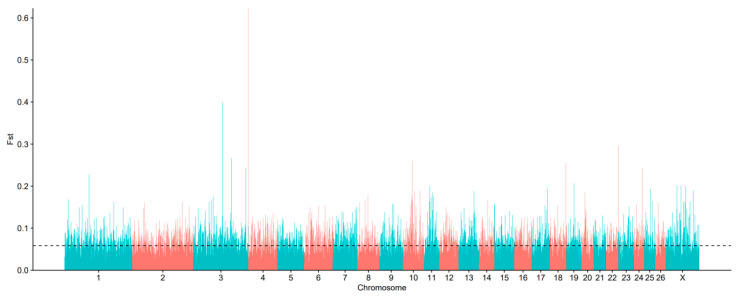
Selection signal analysis. genome-wide distribution of F_ST_ (LLS vs. HS). Note: The *X*-axis is indicative of chromosomal position, whilst the *Y*-axis denotes F_ST_ values. The black dashed line indicates the top 1% significance threshold. Red and blue are used to distinguish chromosomes; for each set interval, the F_ST_ value of different chromosomes.

**Figure 3:**
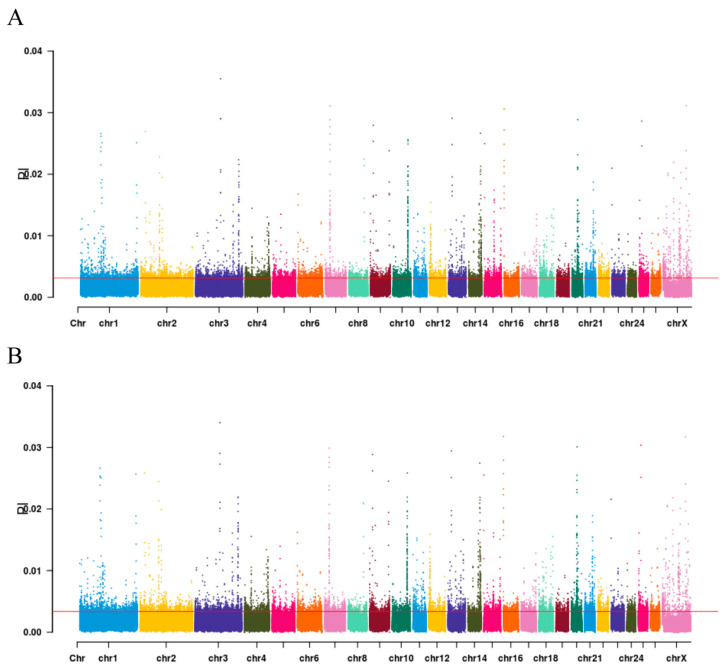
Selection signal analysis. A. π-value results for HS; B. π-value results for LLS. Note: Manhattan plot showing the selected regions of the introduced breeds according to F_ST_ method. The solid red line denotes the top 1% significance threshold, and the data points above the dotted line are the selected regions. Different colors are also used to distinguish chromosomes.

The objective of this study was to identify candidate genomic regions under selection for caudal fat deposition across divergent fat-tailed sheep phenotypes. To this end, this study employed F_ST_-based selection scans, using the top 5% of F_ST_ values as the significance threshold. A total of 78,359,989 single-nucleotide polymorphisms (SNPs) were identified within genomic bins displaying significant population structure differentiation (F_ST_) between LLS and HS populations. As illustrated in Figure 2, the genome-wide distribution of F_ST_ values demonstrates significant heterogeneity in F_ST_ frequency patterns across chromosomes. The highest F_ST_ frequencies were observed on chromosomes 1, 3, 4, 10, 18, 22, and 24, suggesting elevated homozygosity at allelic sites in these regions and potential strong positive selection. Selection peaks on chromosome 1, 3, 4, 10, 18, 22, and 24 coincide with genes implicated in adipogenesis in prior sheep studies. A selection peak on chromosome 1 encompasses the *GLIS1* gene, a pro-adipogenic transcription factor that may promote preadipocyte accumulation and differentiation, thereby influencing caudal fat deposition. This finding aligns with the marked genetic differentiation observed between fat-tailed and thin-tailed sheep populations. Another prominent peak on chromosome 10 contains genes such as *TBX15* and *VRTN*, which are associated with tail morphology and fat deposition. Genome-wide association studies (GWAS) have confirmed their significant links to tail phenotype traits in sheep. Additionally, selection signals on chromosomes 18 and 22 overlap with genes including *JAZF1* and *MC4R*, both known to play important roles in lipid metabolism. *JAZF1* modulates lipid deposition by regulating the expression of lipogenic enzymes such as FAS and ACC, while *MC4R* is involved in energy balance and adiposity. These results correspond closely with known quantitative trait loci (QTL) for traits such as “carcass fat percentage” on chromosomes 10 and 18, supporting the conserved and critical function of these genes in the genetic architecture of ovine tail fat. Biologically, many of these genes are implicated in core adipogenic pathways such as PPAR signaling and ECM-receptor interactions. For instance, both *GLIS1* and *JAZF1* contribute to enhanced caudal adiposity by regulating preadipocyte differentiation and lipid droplet formation. Cross-population comparisons with other sheep breeds, such as Mongolian sheep and Small-tailed Han sheep, further indicate that these selection signals are recurrent in fat-tailed populations, underscoring their potential as key genetic markers for tail fat deposition across diverse breeds. Figure 3 presents the results of the π-value analysis, which identified 3540 SNPs within the top 5% threshold. After annotation, 2654 candidate genes were obtained. The genes that are related to lipid metabolism include the following *ALOX5* (Arachidonate 5-Lipoxygenase), *ALDH1A2* (Aldehyde Dehydrogenase 1 Family Member A2), *AHR* (Aryl Hydrocarbon Receptor), *APOA4* (Apolipoprotein A4), *SREBF1* (Sterol Regulatory Element Binding Transcription Factor 1), *LRRC16A* (Leucine Rich Repeat Containing 16A), etc.

### 3.4. Identification of Signatures of Selection Between Long-Fat-Tailed and Short-Fat-Tailed Sheep Breeds Using XP-EHH Analysis

**Figure 4:**
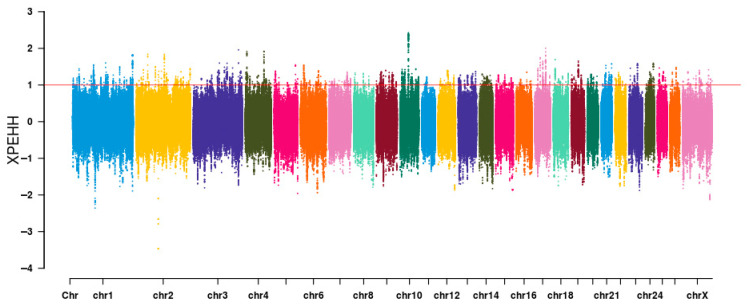
Distribution of selection signals on the autosomes of LLS and HS. Note: Manhattan plot showing the selected regions based on XP-EHH methods between the long-fat-tailed breeds and short-fat-tailed breeds. The *X*-axis is indicative of chromosomal position, whilst the *Y*-axis denotes XP-EHH values. The red dashed line indicates the XP-EHH > 1 significance threshold. Different colors are also used to distinguish chromosomes.

A comparative analysis was performed on the XP-EHH between the LLS and HS groups, which resulted in the identification of genomic regions that were associated with caudal fat deposition. Utilizing LLS as the test population and HS as the reference population, the selection signal analysis revealed distinct genomic signatures, as illustrated in Figure 4. SNPs with XP-EHH > 1 were designated as putatively selected variants, thereby identifying 4985 significant loci. After annotation, 231 candidate genes were obtained. The genes that are related to lipid metabolism include the following *ABCB11* (ATP Binding Cassette Subfamily B Member 11), *CASR* (Calcium Sensing Receptor), *TSHR* (Thyroid Stimulating Hormone Receptor), *SORBS1* (Sorbin and SH3 Domain Containing 1), *CALCR* (Calcitonin Receptor), *GPR39* (G Protein-Coupled Receptor 39), etc. (Table 2).

### 3.5. Identification of Signatures of Selection Between Long-Fat-Tailed and Short-Fat-Tailed Sheep Breeds Using XP-CLR Analysis

**Figure 5:**
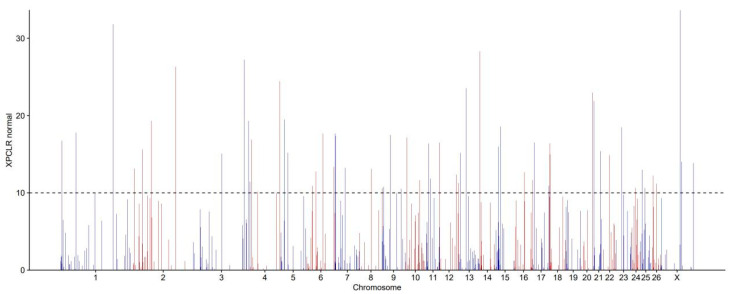
Selection signal analysis. genome-wide distribution of XP-CLR (LLS vs. HS). Note: The *X*-axis is indicative of chromosomal position, whilst the *Y*-axis denotes XP-CLR values. The dashed line indicates the XP-CLR > 10 significance threshold. Different colors are also used to distinguish chromosomes.

The employment of XP-CLR analysis of LLS and HS groups resulted in the identification of 648 sliding windows that were deemed to be under putative selection. As illustrated in Figure 5, the genome-wide distribution of maximum XP-CLR values per window across both populations is presented. Of the sliding windows analyzed, 41 windows with XP-CLR values greater than 10 were designated as putative candidate regions under positive selection (see Table 3). After annotation, 191 candidate genes were obtained. The genes that are related to lipid metabolism include the following *ADCY2* (Adenylyl Cyclase 2), *ITGA11* (Integrin Alpha 11), *ACACA* (Acetyl-CoA Carboxylase Alpha)*, PTPRD* (Protein Tyrosine Phosphatase Receptor Type D), *GPC5* (Glypican 5), etc.

### 3.6. Enrichment Analysis

**Figure 6:**
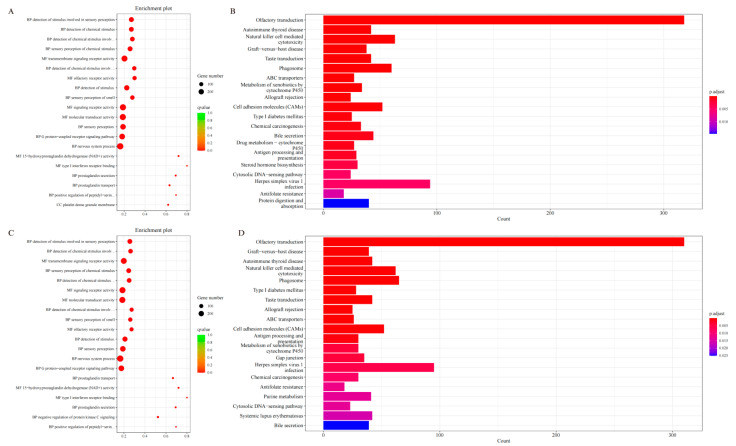
GO enrichment and KEGG enrichment results. (**A**) HS in the first 20 GO enrichment results; (**B**) HS in the KEGG enrichment results; (**C**) LLS in the first 20 GO enrichment results; (**D**) LLS in the KEGG enrichment results.

Significant enrichment analysis of the candidate genes in the LLS group identified 8662 Gene Ontology (GO) biological processes (BP), 1094 cellular components (CC), and 1827 molecular functions (MF) (Figure 6A), as well as 331 Kyoto Encyclopedia of Genes and Genomes (KEGG) pathways (Figure 6B). Integrating the GO and KEGG results subsequently revealed 35 pathways related to metabolism and lipid metabolism. These pathways encompass glycerolipid metabolism, cholesterol metabolism, fatty acid biosynthesis, and the AMPK signaling pathway. In the HS group, candidate genes were significantly enriched in 8786 biological processes (BP), 1052 cellular components (CC), and 1778 molecular functions (MF) (Figure 6C). KEGG enrichment analysis identified 333 significant pathways (Figure 6D). A subsequent investigation into the subject using functional enrichment analysis, based on GO and KEGG terms, identified 35 metabolic pathways associated with lipid metabolism. Of these, the AMPK signaling pathway was the most significantly enriched.

**Figure 7:**
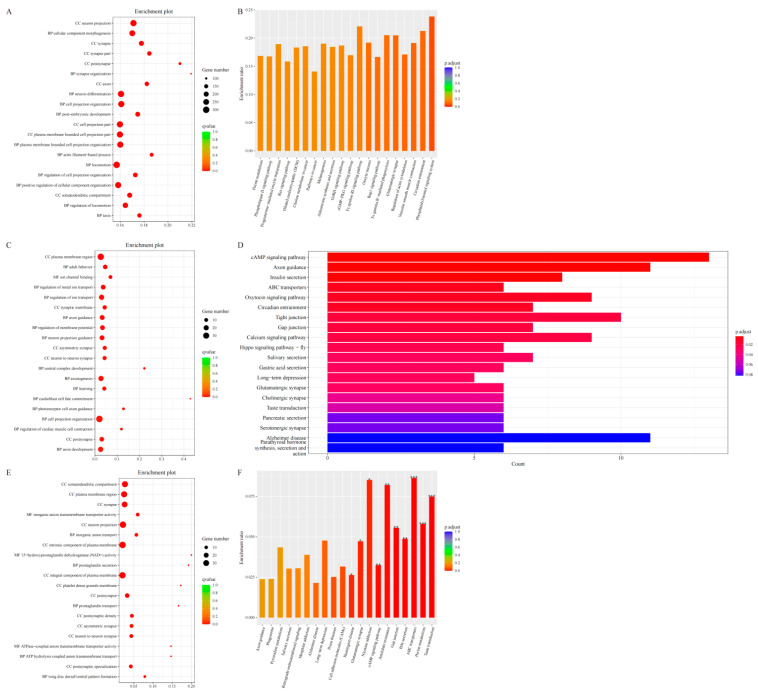
GO enrichment and KEGG enrichment results. (**A**) GO enrichment results of genes screened in the top 20 using the F_ST_ method (LLS vs. HS); (**B**) KEGG enrichment results of genes screened in the top 20 using the F_ST_ method (LLS vs. HS); (**C**) GO enrichment results of genes screened in the top 20 using the XP-EHH method (LLS vs. HS); (**D**) GO enrichment results of genes screened in the top 20 using the XP-EHH method screened genes in KEGG enrichment results (LLS vs. HS); (**E**) GO enrichment results of genes screened in the top 20 using the XP-CLR method (LLS vs. HS); (**F**) KEGG enrichment results of genes screened using the XP-CLR method (LLS vs. HS).

The F_ST_ analysis of the LLS-HS comparison identified 2654 genes under selection for enrichment analysis. These genes were significantly enriched across 29 molecular function (MF) categories, 310 biological process (BP) categories, and 65 cellular component (CC) categories (Figure 7A; Table S2). Prominent enrichments associated with lipid metabolism included the GO terms ‘lipid metabolic process’ (GO:0006629), ‘cellular lipid metabolic process’ (GO:0044255), ‘regulation of lipid biosynthesis’ (GO:0046889) and ‘regulation of lipid metabolic process’ (GO:0019216) (Figure 7B). Using XP-EHH analysis, we detected significant enrichment of candidate genes in 662 molecular functions, 3828 biological processes, and 468 cellular components (Figure 7C; Table S2). Prominent enrichments in lipid metabolism pathways included the peroxisome proliferator-activated receptor (PPAR) signaling pathway, bile secretion, linoleic acid metabolism, and adipocytokine signaling pathway (Figure 7D). Using the XP-CLR method, we identified candidate genes that were significantly enriched in 21 molecular functions, 31 biological processes, and 24 cellular components (Figure 7E; Table S2). Further KEGG analysis demonstrated the enrichment of these selected genes in 565 pathways, with a particular focus on lipid metabolism pathways, such as cAMP signaling, PPAR signaling, PI3K-Akt signaling, TGF-β signaling, AMPK signaling, and Wnt/β-catenin signaling (Figure 7F).

### 3.7. Shared Gene

**Figure 8:**
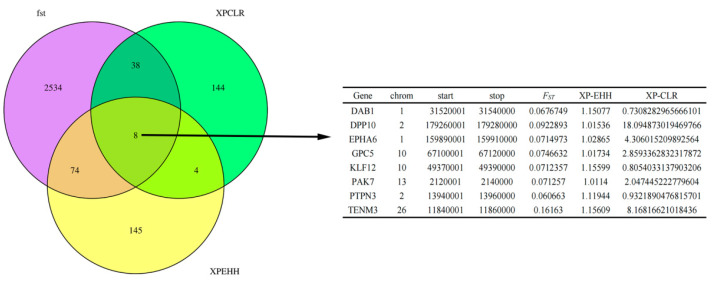
Share the results of gene enrichment analyses. Note: Venn diagram showing the overlapped genes from the F_ST_, XP-EHH, and XP-CLR analyses.

A Venn diagram analysis of the selection-related genomic regions identified in the LLS and HS populations, using the F_ST_, XP-EHH, and XP-CLR methods, revealed substantial overlap. Specifically, 46 candidate genes were shared between F_ST_ and XP-CLR, while 82 were shared between F_ST_ and XP-EHH. There was minimal overlap between XP-EHH and XP-CLR, with only 12 genes shared between the two methods. Eight candidate genes were common to all three methods: *DAB1* (Disabled 1 Adaptor Protein), *DPP10* (Dipeptidyl Peptidase 10), *EPHA6* (EPH Receptor A6), *GPC5* (Glypican 5), *KLF12* (Kruppel-Like Factor 12), *PAK7* (p21-Activated Kinase 7), *PTPN3* (Protein Tyrosine Phosphatase Non-Receptor Type 3), and *TENM3* (Tenascin-M Like 3) (Figure 8). *DAB1* promotes the differentiation of preadipocytes into mature adipocytes by activating the P13K-AKT pathway, which phosphorylates and inhibits *GSK3β* (glycogen synthase kinase 3β), thereby relieving *GSK3β*-mediated suppression of *PPARγ*—a master regulator of adipogenesis. Moreover, *DAB1* suppresses the NF-κB signaling pathway, reducing the secretion of pro-inflammatory cytokines such as *TNF-α* and *IL-6* in adipose tissue, thus preventing inflammation-driven aberrant lipid accumulation. *DPP10* binds to β-adrenergic receptors on adipocyte membranes, inhibiting epinephrine-induced cAMP-PKA signaling. This suppression reduces phosphorylation of *HSL* (hormone-sensitive lipase), a key enzyme in triglyceride hydrolysis. Consequently, lipolysis is impaired, promoting triglyceride storage. Overexpression of *DPP10* in human adipocytes has been shown to reduce lipolysis rates by 25–30%. *EPHA6*, upon binding its ligand Ephrin-A1, activates the Ras-MAPK pathway and facilitates directed migration of preadipocytes. Its expression is negatively correlated with wool fiber diameter; individuals with higher *EPHA6* expression produce finer wool fibers. *GPC5* expression influences energy partitioning in sheep: high expression promotes the conversion of excess energy into caudal fat under high-calorie diets, whereas low expression favors muscle growth. This makes *GPC5* a potential molecular marker for selecting between fat- and meat-oriented breeding strategies. *KLF12* is more highly expressed in visceral fat than in caudal fat and may inhibit visceral lipid synthesis, preventing excessive ectopic fat deposition. *PAK7* is expressed in the liver, muscle, and adipose tissues of sheep, and its expression level is positively correlated with daily weight gain. Studies of Tan sheep showed that individuals with high *PAK7* expression exhibited 15–20% higher average daily gain and improved feed efficiency. Under different nutritional regimens, *PTPN3* expression is upregulated in ovine adipose tissue during high-energy intake, possibly attenuating insulin signaling to prevent excessive fat deposition. Conversely, aberrant *PTPN3* expression is associated with insulin resistance and reduced caudal fat accumulation. *TENM3* is highly expressed in the outer root sheath of sheep hair follicles. It regulates extracellular matrix (ECM) remodeling, influencing hair shaft growth and structure. In Merino sheep, *TENM3* expression is positively correlated with wool fiber strength, with higher expression resulting in greater resistance to breakage. Identified genes such as PPARγ and GPC5 can serve as targets for marker-assisted selection to preserve fat-tail traits in LLS.

## 4. Discussion

Through whole-genome resequencing analysis, substantial genetic divergence was revealed between Lanzhou Fat-Tailed sheep and Hu sheep. Selection signature analyses—including F_ST_, XP-EHH, and XP-CLR, detected signals of selection across multiple genomic regions associated with biological processes such as growth and development, metabolic regulation, and immune response [24,25]. For instance, putatively selected genes related to lipid metabolism and meat quality (e.g., those involved in AMPK and PPAR signaling pathways) were identified in LLS, consistent with its dual-purpose meat-and-fat characteristics [26]. In contrast, HS exhibited significant selection signals near genes influencing reproductive performance (e.g., BMPR1B), supporting its genetic propensity for high fecundity. These findings illuminate the genetic mechanisms underlying environmental adaptation and trait selection in distinct sheep breeds [27].

While PCA was performed, the limited sample size (n = 15 per group) constrained robust population structure inference. Therefore, some detected selection signals may be influenced by residual population stratification or genetic drift. These limitations are acknowledged and will be addressed in future studies using larger cohorts and replication analyses.

Population genetic analyses revealed significant selection signals in the DAB1 gene region between Lanzhou Fat-Tailed sheep and Hu sheep. DAB1, a key adaptor protein in the Reelin signaling pathway, mediates neuronal migration termination through tyrosine phosphorylation and regulates cortical laminar organization and synaptogenesis. Its functions are primarily associated with neural development, with no established direct link to fat deposition [27,28]. GPC5, a heparan sulfate proteoglycan, negatively regulates adipogenesis by competitively binding Wnt3a and inhibiting β-catenin nuclear translocation [29]. In Lanzhou Fat-Tailed sheep, methylation analysis showed hypermethylation in exonic regions of GPC5, potentially reducing its expression by inhibiting transcriptional elongation and thereby promoting fat deposition [30]. KLF12 acts as a key negative regulator of adipogenesis by directly binding to the PPARγ promoter and suppressing its transcription. PAK7, a member of the p21-activated kinase family, promotes cell invasion in gastric and osteosarcoma contexts via the Rac1/Cdc42 pathway. Although no direct association with fat deposition has been reported, hypomethylation in the PAK7 promoter region may enhance its transcriptional activity. Notably, a high observed heterozygosity (Ho = 0.692) at this locus in the Hu sheep population suggests balancing selection, potentially related to reproductive or immune adaptation [31,32].

Cross-population selection analysis (F_ST_/XP-EHH/XP-CLR) revealed eight genes to be shared by Lanzhou fat-tailed sheep and Hu sheep. Functional enrichment analysis (GO/KEGG) revealed that *DAB1* and *GPC5* exhibit unique synergistic potential in regulating lipid metabolism by modulating lipid synthase activity, adipogenic differentiation pathways, and fatty acid metabolism gene networks simultaneously.

The phosphotyrosine-binding (PTB) domain of the *DAB1* gene specifically recognizes the head group of phosphatidylinositol 4,5-bisphosphate (PtdIns(4,5)P_2_) via its positively charged surface region and interacts with receptors such as *ApoER2* and *VLDLR*, thereby participating in phosphorylation events within the Reelin signaling pathway [33,34,35]. Mutations in the lipid-binding site of this domain markedly impair *DAB1* localization to the plasma membrane and inhibit activation of the downstream PI3K/Akt pathway [33,34]. Transcriptomic studies in fat-tailed sheep breeds have identified *DAB1* as a candidate gene linked to lipid metabolism, potentially promoting adipogenesis through the PI3K/AKT signaling pathway, with a likely role in regulating caudal fat deposition [36]. Impaired membrane localization of mutated *DAB1* suppresses PI3K/AKT signaling and disrupts lipid metabolism. Upon phosphorylation, *DAB1* activates PI3K, leading to Akt activation, which enhances lipogenic enzyme activity via *GSK3*β inhibition, thereby promoting lipid storage [33,37,38]. Furthermore, *DAB1* regulates cell migration through the Crk/C3G/Rap1 pathway, which may influence the positioning and differentiation of adipocyte precursors and contribute to the development and morphological patterning of caudal adipose tissue [33,39]. In summary, *DAB1* is regarded as a key lipid metabolism-associated gene in sheep, particularly in the regulation of tail fat phenotypes in fat-tailed breeds [36].

*GPC5*, a heparan sulfate proteoglycan (HSPG), competitively binds to Wnt3a and inhibits β-catenin nuclear translocation, thereby downregulating the expression of adipogenic genes such as PPARγ and C/EBPα and ultimately suppressing adipocyte differentiation [40,41]. In ovine caudal adipose tissue, elevated *GPC5* expression is closely associated with PPARγ signaling enrichment and may influence lipid deposition by modulating PPARγ target genes such as *FABP4* and *ADIPOQ*, suggesting a potential regulatory role in tail fat accumulation [6,42,43]. Furthermore, *GPC5* may contribute to lipid droplet stability and suppress lipolysis by influencing the function of Perilipin family proteins, including *Plin5*. *Plin5* inhibits lipolysis by binding to the *ATGL*/*CGI*-*58* complex, thereby reducing free fatty acid release and alleviating lipotoxicity, which helps maintain adipose tissue homeostasis [44,45]. Notably, *GPC5* expression is significantly higher in the adipose tissue of Guangling Large-Tailed sheep compared to thin-tailed breeds and is associated with the ECM-receptor interaction pathway, suggesting its potential role in promoting adipocyte hypertrophy through extracellular matrix remodeling and thereby influencing caudal fat development and morphology [44,46]. In summary, *GPC5* plays a critical role in ovine adipogenesis and tail fat phenotype regulation, primarily through suppressing Wnt signaling and modulating lipid metabolic processes.

In terms of regulating lipid metabolism, *DAB1* primarily drives lipid synthesis via the Reelin-PI3K pathway, while *GPC5* modulates adipocyte homeostasis by regulating ion channels. Lanzhou fat-tailed sheep exhibit an increased capacity for lipid storage, which is due to elevated *DAB1* expression and enhanced *GPC5* activity. This metabolic adaptation is evident in the form of >36% unsaturated fatty acid content in tail adipose tissue. In contrast, Hu sheep exhibit a different metabolic profile characterized by *GPC5* polymorphisms that favor immunometabolic trade-offs, prioritizing basal physiological maintenance over lipid accumulation [42]. Divergent tail morphogenesis is manifested through breed-specific molecular regulation. For example, Lanzhou fat-tailed sheep develop elongated tails via *DAB1*/*GPC5*-mediated adipocyte hyperplasia/hypertrophy, resulting in adipocytes that are 40–50% larger than those of Hu sheep. In contrast, Hu sheep’s compact tails arise from *GPC5* polymorphism-induced membrane inhibition and reductions in *DAB1* phosphorylation that predispose to lipolysis [47].

## 5. Conclusions

The present study employed selection signature analysis to investigate the genetic basis of divergent caudal fat deposition between Lanzhou Fat-Tailed and Hu sheep. A total of eight candidate genes under selection were identified, with DAB1 and GPC5 proving to be promising candidate targets associated with caudal lipid metabolism. However, limitations such as the small sample size and lack of gene expression validation should be addressed in future studies to confirm the functional relevance of these candidate genes. These findings provide crucial insights for the conservation and utilization of genetic resources in indigenous Chinese breeds of livestock. Next steps will include validation via gene expression or CRISPR functional studies.

## Figures and Tables

**Table 1:** Sample information.

Sample	Abbreviation	Size	Type	Photo
Lanzhou fat-tailed sheep	LLS	15	blood	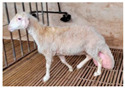
Hu sheep	HS	15	blood	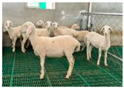

**Table 2:** Candidate regions under positive selection identified in the genomes of LLS and HS.

Chromosomes	N_Variants	Bin_Start	Bin_End	Gene
chr1	234	99950001	99970000	*LOC101114737*
chr3	337	119610001	119630000	
chr3	153	119650001	119670000	
chr3	128	119660001	119680000	
chr3	248	119670001	119690000	
chr3	212	154030001	154050000	
chr3	206	213100001	213120000	
chr3	206	213110001	213130000	
chr4	195	160001	180000	
chr4	121	170001	190000	
chr4	84	180001	200000	
chr4	63	190001	210000	
chr4	42	200001	220000	
chr10	323	38570001	38590000	
chr10	355	38580001	38600000	
chr10	334	38590001	38610000	
chr10	290	38600001	38620000	
chr10	328	38610001	38630000	
chr10	398	38620001	38640000	
chr18	149	66460001	66480000	*TRNAE-UUC-84*, *LOC101104530*
chr18	212	66470001	66490000	*LOC105603275*, *LOC105603310*
chr18	241	66480001	66500000	*LOC105603275*, *LOC105603310*, *LOC106990142*
chr18	188	66490001	66510000	*LOC105603275*, *LOC106990142*
chr19	242	31750001	31770000	*MITF*
chr22	1	50780001	50800000	
chr24	324	35120001	35140000	*COL26A1*
chr24	225	35130001	35150000	*COL26A1*
chrX	145	43220001	43240000	*ZNF674*
chrX	147	60010001	60030000	*TEX11*
chrX	152	79200001	79220000	*FATE1*, *CNGA2*

**Table 3:** Candidate regions subject to positive selection on the genome of HS over LLS identified by XP-CLR test.

Chromosomes	Start	Stop	XP-CLR	Chromosomes	Start	Stop	XP-CLR
1	205504001	205506000	21.286	12	595001	597000	14.876
1	1205503001	205505000	12.206	12	65394001	65396000	11.191
1	59281001	59283000	11.906	12	594001	596000	10.862
1	3687001	3689000	11.216	12	74271001	74273000	10.209
2	179123001	179125000	18.095	13	29303001	29305000	19.179
2	82793001	82795000	13.294	13	7107001	7109000	12.393
3	200422001	200424000	15.017	16	38115001	38117000	10.056
3	217141001	217143000	10.660	18	3797001	3799000	19.272
6	67238001	67240000	24.500	18	4993001	4995000	17.599
6	110111001	110113000	18.562	18	3556001	3558000	11.217
6	40289001	40291000	17.723	18	55897001	55899000	11.204
6	27372001	27374000	15.127	20	47588001	47590000	16.284
6	25664001	25666000	10.685	21	6528001	6530000	12.379
6	113967001	113969000	10.321	22	23001001	23003000	14.462
7	2631001	2633000	16.668	22	23005001	23007000	12.506
7	4248001	4250000	16.384	23	24097001	24099000	11.478
7	41107001	41109000	12.502	25	8859001	8861000	11.271
8	46959001	46961000	12.301	26	8807001	8809000	12.374
10	7466001	7468000	14.839	26	21757001	21759000	11.324
10	57489001	57491000	10.097	X	76736001	76738000	21.189
11	15145001	15147000	11.448				

## Data Availability

All data generated during the current study is included in this manuscript.

## Supplementary Materials

The following supporting information can be downloaded at: https://www.mdpi.com/article/10.3390/ani15203046/s1, Table S1: Summary statistics of whole-genome resequencing datasets from LLS and HS.
