# Supplementary material for: Whole-Genome Resequencing Identifies Candidate Genes for Tail Fat Deposition in Sheep
Source: Animals (Basel). 2025 Oct 20;15(20):3046. doi: 10.3390/ani15203046 (PMC12561157; doi:10.3390/ani15203046)
Supplement: Supplementary file 1 [file animals-15-03046-s001.zip › animals-3842697-supplementary.pdf]

Supplementary Table

Table S1. Summary statistics of whole-genome resequencing datasets from LLS and HS.

Sample Name	Raw Base(G)	Clean reads	Mapped reads	Mapping rate	Q20 (%)	Q30 (%)	GC Content (%)
LLS 1	36485799600	241262750	240495591	99.68%	98.46%	95.26%	47.76%
LLS 2	34338341100	227037246	226327500	99.69%	98.21%	94.47%	44.46%
LLS 3	34619694000	228763214	227885997	99.62%	98.24%	94.54%	46.81%
LLS 4	30953311500	204328674	203469941	99.58%	98.01%	93.86%	48.20%
LLS 5	30227679600	199686058	198982164	99.65%	97.96%	93.66%	47.50%
LLS 6	33988748100	224879578	224218600	99.71%	98.30%	94.71%	44.92%
LLS 7	31841494800	210086292	209277739	99.62%	97.83%	93.41%	46.68%
LLS 8	30854486400	203773508	203092347	99.67%	98.02%	93.85%	45.70%
LLS 9	39095427900	258162034	257267020	99.65%	98.05%	93.98%	45.50%
LLS 10	32832870900	216722690	215938607	99.64%	97.82%	93.43%	45.05%
LLS 11	30948163200	204165428	203327333	99.59%	97.87%	93.43%	47.03%
LLS 12	30996684000	204704766	203957896	99.64%	98.01%	93.83%	47.22%
LLS 13	29915011800	197543552	196858255	99.65%	97.97%	93.76%	45.61%
LLS 14	30039834600	198289082	197586297	99.65%	97.90%	93.53%	45.84%
LLS 15	41431522500	274692624	273965553	99.74%	97.86%	93.03%	47.34%
HS 1	33872780400	223102876	222326749	99.65%	97.52%	92.15%	45.97%
HS 2	38864684100	256061626	255121334	99.63%	97.69%	92.74%	46.96%
HS 3	37876964700	251192890	250661546	99.79%	97.95%	93.27%	44.83%
HS 4	30689370300	202181164	201417855	99.62%	97.53%	92.22%	47.51%
HS 5	64426462500	424425386	422848153	99.63%	97.61%	92.45%	47.47%
HS 6	51274744200	339798022	338924761	99.74%	97.53%	91.76%	49.46%
HS 7	39931808400	264272414	263540829	99.72%	97.83%	93.19%	46.88%
HS 8	29846307600	197282224	196673479	99.69%	98.00%	93.61%	46.59%
HS 9	42524391900	281030818	280137541	99.68%	97.53%	92.30%	44.17%
HS 10	39768764400	263047330	262244943	99.69%	97.87%	93.28%	47.79%
HS 11	42381682500	278859904	277714692	99.59%	97.61%	92.56%	44.93%
HS 12	40362747900	265943824	264931304	99.62%	97.55%	92.31%	46.65%
HS 13	45964625700	302690638	301592696	99.64%	97.66%	92.66%	46.44%
HS 14	40779804300	268987598	267890743	99.59%	97.47%	92.18%	47.75%
HS 15	54037123200	356317224	355051803	99.64%	97.59%	92.36%	46.89%

Table S2.

ID	pvalue	qvalue	Count
GO:0006629	0.355470719090858	0.635965009781946	152
GO:0044255	0.324052200097673	0.605687873141307	126
GO:0046889	0.379593659549641	0.635965009781946	16
GO:0019216	0.316382757904207	0.604014349403161	64
GO:0045721	0.491558676260588	0.70038795105065	2
GO:0045444	0.180545814349956	0.529605683310326	18
GO:0006641	0.235620434263468	0.554535146660955	14
GO:0045722	0.192336872817982	0.529605683310326	4
GO:0060394	0.182298895792771	0.529605683310326	3
GO:0055119	0.182298895792771	0.529605683310326	3
GO:0034389	0.40643576988261	0.652991140602631	4
GO:0008610	0.508945318097256	0.705207396841314	75
GO:0006631	0.437304039017987	0.67319408345091	42
GO:0016324	0.477439383130203	0.69294228109911	51
GO:0016325	0.0929788960554797	0.432006265551764	8
GO:0016051	0.351162010206717	0.635965009781946	17
GO:0006644	0.00965542748272076	0.15262503867445	66
GO:0032787	0.360589596017714	0.635965009781946	67
GO:0016323	0.000200281402114794	0.0178212320403601	12
GO:0016324	0.00161457170516443	0.0453175088987516	12
GO:0032941	0.175459360439549	0.338776322605982	1
GO:0060612	0.106475253602168	0.281295463351672	2
GO:0045598	0.403601097924524	0.492759057943617	2
GO:0010884	0.392417245628416	0.613632185883748	1
GO:0006633	0.711725101122144	0.801184271693636	1
